# γδ T cell-intrinsic IL-1R promotes survival during *Staphylococcus aureus* bacteremia

**DOI:** 10.3389/fimmu.2023.1171934

**Published:** 2023-07-07

**Authors:** Yu Wang, Michael Z. Ahmadi, Dustin A. Dikeman, Christine Youn, Nathan K. Archer

**Affiliations:** Department of Dermatology, Johns Hopkins University School of Medicine, Baltimore, MD, United States

**Keywords:** *Staphylococcus aureus*, IL-1R, bacteremia, T cells, host defense, cytokines

## Abstract

*Staphylococcus aureus* is a leading cause of bacteremia, further complicated by the emergence of antibiotic-resistant strains such as methicillin-resistant *S. aureus* (MRSA). A better understanding of host defense mechanisms is needed for the development of host-directed therapies as an alternative approach to antibiotics. The levels of IL-1, IL-17, and TNF-α cytokines in circulation have been associated with predictive outcomes in patients with *S. aureus* bacteremia. However, their causative role in survival and the cell types involved in these responses during bacteremia is not entirely clear. Using a mouse model of *S. aureus* bacteremia, we demonstrated that IL-17A/F and TNF-α had no significant impact on survival, whereas IL-1R signaling was critical for survival during *S. aureus* bacteremia. Furthermore, we identified that T cells, but not neutrophils, monocytes/macrophages, or endothelial cells were the crucial cell type for IL-1R-mediated survival against *S. aureus* bacteremia. Finally, we determined that the expression of IL-1R on γδ T cell, but not CD4^+^ or CD8^+^ T cells was responsible for survival against the *S. aureus* bacteremia. Taken together, we uncovered a role for IL-1R, but not IL-17A/F and TNF-α in protection against *S. aureus* bacteremia. Importantly, γδ T cell-intrinsic expression of IL-1R was crucial for survival, but not on other immune cells or endothelial cells. These findings reveal potential cellular and immunological targets for host-directed therapies for improved outcomes against *S. aureus* bacteremia.

## 1 Introduction

*Staphylococcus aureus* is a leading cause of bacteremia (1), with a mortality rate of ~25% due to the emergence of antibiotic-resistant strains such as methicillin-resistant *S. aureus* (MRSA) (2). Furthermore, all vaccines to date have failed in clinical trials against *S. aureus* invasive infections (3, 4). Thus, a better understanding of host defense mechanisms is needed for the development of host-directed therapies as an alternative approach to antibiotics.

The IL-1, IL-17, and TNF-α cytokines have been implicated in host defense against *S. aureus* skin and orthopedic infections (5–8). Moreover, IL-1, IL-17, and TNF-α cytokine levels in circulation have been associated with predictive outcomes in patients with *S. aureus* bacteremia (4, 9–12). For instance, elevated IL-1β at the time of patient admission correlated with reduced duration of the *S. aureus* bacteremia (11). However, whether the IL-1, IL-17, and TNF-α cytokines have a causative role in host survival and the cell types involved in these responses during *S. aureus* bacteremia is not entirely clear.

In this study, we evaluated the contributions of IL-1α/β, IL-17A/F, and TNF-α to host survival during *S. aureus* bacteremia using a preclinical mouse model. Furthermore, we identified the specific cell types that promote host survival using mice with specific deletion of IL-1R on T cells, myeloid cells, neutrophils, and endothelial cells.

## 2 Materials and methods

### 2.1 Bacterial preparation

The community-acquired methicillin-resistant *S. aureus* (MRSA) USA300 SF8300 strain, a kind gift from Dr. Binh Diep (UCSF), was cultured in tryptic soy broth (TSB) as previously described (13, 14). Briefly, SF8300 was streaked onto a tryptic soy agar (TSA) plate (TSB plus 1.5% bacto agar (BD Biosciences)) and grown overnight at 37°C in a bacterial incubator. Two to three single colonies were picked and cultured in TSB at 37°C in a shaking incubator (240 rpm) overnight (18 h), followed by a 1:50 subculture at 37°C for 2 h to obtain mid-logarithmic phase bacteria. The bacteria were pelleted, washed 3 times with sterile PBS, resuspended in sterile freezing medium (10% glycerol in sterile PBS) at a concentration of 1×10^10^ CFU/ml and aliquots stored in cryovials at -80°C until needed. The number of CFUs was confirmed with overnight culture on TSA plates.

### 2.2 Mice

Age-matched 6-8-week-old female mice on C57BL/6 background were used for all experiments. The IL-1α^–/–^, IL-1β^–/–^, and IL-17A/F^–/–^ mice were provided by Dr. Yoichiro Iwakura (University of Tokyo). The VE-Cad^Cre^×IL-1R^fl/fl^ (VE-Cad-IL-1R^–/–^) mice, which lack IL-1R signaling in endothelial cells were provided by Dr. Michael O’Connell (NIH/NIAID). WT C57BL/6, TNF-α^–/–^ (B6.129S-tnf^tm1Gkl^/J), IL-1R^–/–^ (B6.129S7-l1r1^tm1Imx^/J), Lck^Cre^ (B6.Cg-Tg(Lck-cre)548Jxm/J), LysM^Cre^ (B6.129P2-Lyz2^tm1(cre)Ifo^/J), CD4^Cre^ (Tg(Cd4-cre)1Cwi/BfluJ), S100A8^Cre^ (B6.Cg-Tg(S100A8-cre,-EGFP)1Ilw/J), TCRδ^CreER^ (B6.129S-Tcrd^tm1.1(cre/ERT2)Zhu^/J), and IL-1R^fl/fl^ mice (B6.129(Cg)-Il1r1^tm1.1Rbl^/J) were obtained from Jackson Laboratories (Bar Harbor, ME).

Lck^Cre^ mice were crossed with IL-1R^fl/fl^ mice to obtain Lck^Cre^×IL-1R^fl/fl^ (Lck-IL-1R^–/–^), which lack IL-1R signaling in pan-T cells. LysM^Cre^ were crossed with IL-1R^fl/fl^ mice to obtain LysM^Cre^×IL-1R^fl/fl^ (LysM-IL-1R^–/–^) mice, which lack IL-1R signaling in myeloid cells. S100A8^Cre^ were crossed with IL-1R^fl/fl^ mice to obtain S100A8^Cre^×IL1R^fl/fl^ (S100A8-IL-1R^–/–^) mice, which lack IL-1R signaling in neutrophils. CD4^Cre^ were crossed with IL-1R^fl/fl^ mice to obtain CD4^Cre^×IL1R^fl/fl^ (CD4-IL-1R^–/–^) mice, which lack IL-1R signaling in CD4-expressing cells, including both CD4^+^ and CD8^+^ T cells (due to dual expression of CD4 in both T cell types during thymic development). TCRδ^CreER^ mice were crossed with IL-1R^fl/fl^ mice to obtain TCRδ^CreER^×IL1R^fl/fl^ (TCRδ-IL-1R^–/–^) mice, which lack IL-1R signaling in γδ T cells upon tamoxifen-inducible deletion.

### 2.3 Study approval

All mouse strains were bred and maintained under the same specific pathogen-free conditions, with air-isolated cages at an American Association for the Accreditation of Laboratory Animal Care (AAALAC)-accredited animal facility at Johns Hopkins University and handled according to procedures described in the Guide for the Care and Use of Laboratory Animals as well as Johns Hopkins University’s policies and procedures as outlined in the Johns Hopkins University Animal Care and Use Training Manual. This study was approved by the Johns Hopkins Animal Care and Use Committee (Protocol #: MO21M378).

### 2.4 Intravenous infection

The *S. aureus* bacteremia model was modified from previously described protocols (15, 16). Briefly, 6-to-8-week-old female C57BL/6 mice were anesthetized (inhalation of 2% isoflurane) and inoculated intravenously with 4.8-5.8 × 10^7^ SF8300 in a 100-μL volume of PBS using a 29-gauge insulin syringe *via* the retro-orbital vein to achieve an LD90.

### 2.5 Tamoxifen-inducible deletion of IL-1R

The inducible deletion of IL-1R on γδ T cells was modified from a previously described protocol (17). The TCRδ-IL-1R^–/–^ mice were treated daily with 100 μl of 1 mg/ml tamoxifen in sunflower oil injected intraperitoneally for 5 consecutive days. The bacteremia infections were performed 10 days after the last tamoxifen injection. Wild-type (WT) mice were subjected to the same tamoxifen regimen when paired with TCRδ-IL-1R^–/–^ mice. Tamoxifen-inducible deletion of IL-1R was confirmed by flow cytometry, which was comparable to the ~60% deletion efficiency in γδ T cells in TCRδ^creER^ mice based on prior reports (18).

### 2.6 Flow cytometry

For flow cytometric analysis, 100 μl of peripheral blood and spleen was collected from tamoxifen-treated WT and TCRδ-IL-1R^–/–^ mice 3h after intravenous infection. Red blood cells were lysed with ACK lysis buffer (ThermoFisher Scientific) and cells were resuspended in FACS buffer (PBS containing 1% BSA and 2mM EDTA). Spleen was manually pushed through a cell separation filter (40 µm) and resuspended in FACS buffer. Single cell suspensions were stained for viability (Viobility 405/520 viability kit, Miltenyi Biotec) and TruStain fcX (Biolegend) was used to block Fc receptor binding. Next, blood single cells were surface stained with the following mAbs: PE-Vio770-CD3 (REA641, Miltenyi Biotec), PE-CD8a (REA601, Miltenyi Biote), APC-Vio770-CD4 (REA604, Miltenyi Biote) VioBlue-TCRγδ (REA633, Miltenyi Biotec), and APC-CD121α (clone JAMA-147, BioLegend). The γδ T cells were identified as CD3^+^CD4^-^CD8^-^TCRγδ^+^ cells from the live cell population. Spleen single cells were surface stained with the following mAbs: PerCP-Vio700-CD45 (REA737, Miltenyi Biotec), APC-CD11b (REA592, Miltenyi Biotec), VioBlue-Ly6C (REA796, Miltenyi Biotec), APC-Vio770-Ly6G (REA526, Miltenyi Biotec), and PE-Vio770-F4/80 (REA126, Miltenyi Biotec). Cell acquisition was performed on a MACSQuant analyzer (Miltenyi Biotec) and data analyzed using MACSQuantify software (Miltenyi Biotec). See **Supplementary Figure 1** for gating strategy.

### 2.7 Ex vivo CFU enumeration

At 3h post infection, mice were euthanized, and the spleen, liver, and kidneys were harvested and ex vivo CFU were isolated as previously described (5, 19). The tissue specimens were homogenized (PRO200 Series homogenizer; PRO Scientific) and then serially diluted and cultured overnight on TSA plates at 37°C. Ex vivo CFU from the homogenized tissue were then enumerated from the plates.

### 2.8 Statistical analyses

Survival rates were compared by log rank (Mantel-Cox) test and data from single comparisons analyzed by Student’s t test (two-tailed), as indicated in the figure legends. All statistical analyses were calculated with Prism software (GraphPad 9.5 Software, La Jolla, California). CFU data are presented as geometric mean ± geometric standard deviation (SD). All other data are presented as mean ± standard error of the mean (SEM) and values of *P <*0.05 were considered statistically significant.

## 3 Results

### 3.1 IL-1R signaling improves survival during *S. aureus* bacteremia

**Figure 1:**
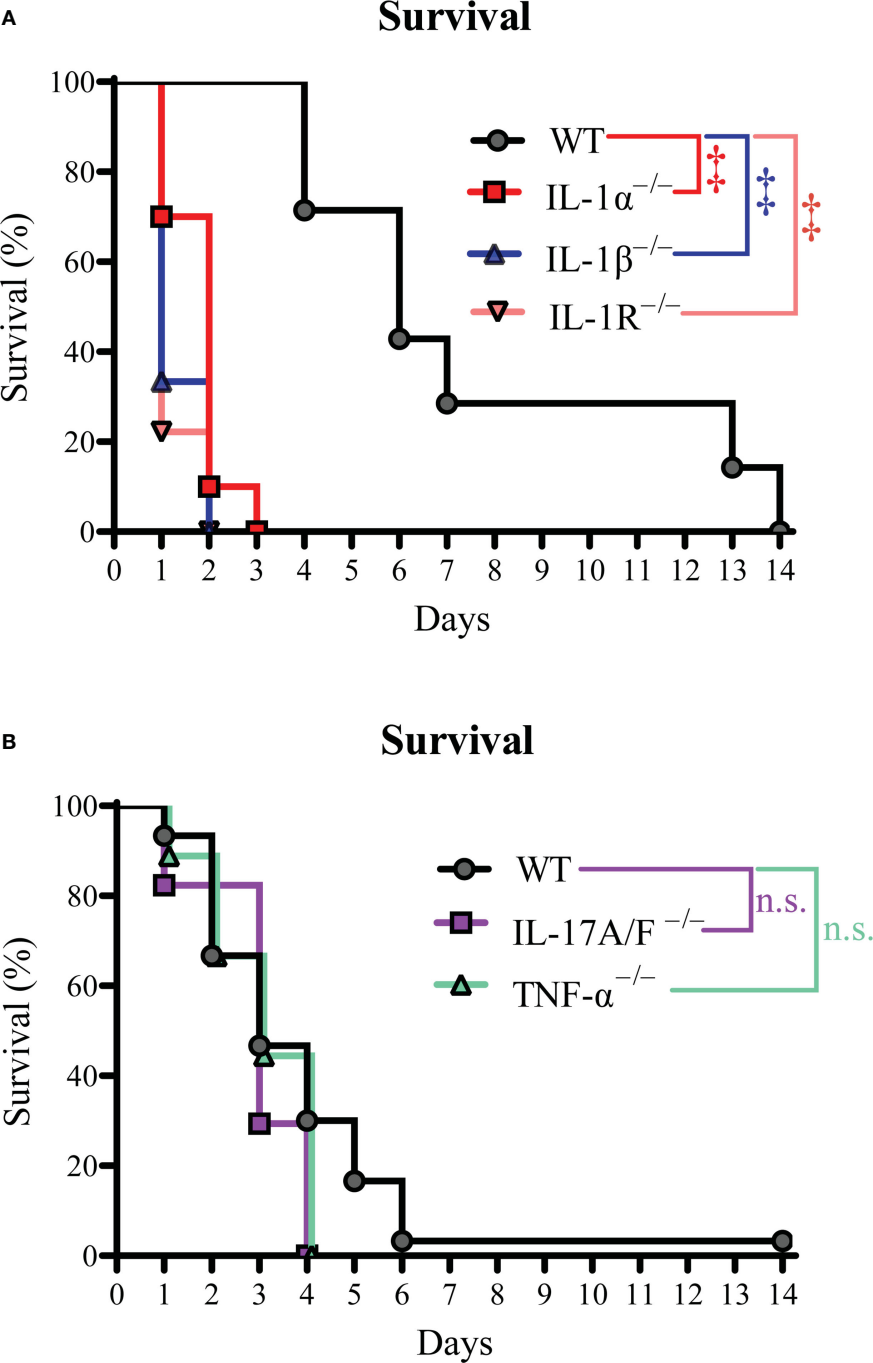
IL-1R signaling improves survival during *S. aureus* bacteremia. The *S. aureus* bacteremia infection was performed on WT, IL-1α^–/–^, IL-1β^–/–^, IL-1R^–/–^, IL-17A/F^–/–^, and TNF-α^–/–^ mice. **(A)** Survival (%) of WT, IL-1α^–/–^, IL-1β^–/–^, and IL-1R^–/–^ mice (n=7-10 per group, average inoculum = 4.8 × 10^7^ CFUs). **(B)** Survival (%) of WT, IL-17A/F^–/–^, and TNF-α^–/–^ mice (n=9-30 per group, average inoculum = 5.4 × 10^7^ CFUs). ‡*P* < 0.001 and n.s. = not significant; WT versus IL-1α^–/–^, IL-1β^–/–^, IL-1R^–/–^, IL-17A/F^–/–^, and TNF-α^–/–^ as calculated by log rank (Mantel-Cox) test. Data were combined from at least 2 independent experiments.

The levels of IL-1, IL-17, and TNF-α cytokines in circulation have been associated with predictive outcomes in patients with *S. aureus* bacteremia (4, 9–12). Therefore, we set out to determine the mechanistic effect of IL-1α/β, TNF-α, and IL-17A/F on survival during *S. aureus* bacteremia using a preclinical mouse model whereby 4.8-5.8 × 10^7^ CFUs of *S. aureus* USA300 (SF8300) were injected i.v. and survival measured over time (15, 16). To determine the role of IL-1R signaling, we first performed our bacteremia model on wild-type (WT) C57BL/6 and IL-1R^–/–^ mice and found that IL-1R^–/–^ mice had a statistically significant decrease in survival compared to WT mice (**Figure 1A**). Since IL-1α and IL-1β signal through the IL-1R (20), we next tested IL-1α^–/–^ and IL-1β^–/–^ mice and discovered that both IL-1α^–/–^ and IL-1β^–/–^ mice had a markedly reduced survival compared to WT mice (**Figure 1A**). Next, we examined IL-17A/F^–/–^ and TNF-α ^–/–^ mice and found no statistically significant differences compared to WT mice (**Figure 1B**). Taken together, our data indicated that IL-1α and IL-1β signaling *via* IL-1R enhanced survival during *S. aureus* bacteremia infections.

### 3.2 γδ T cell-intrinsic IL-1R signaling promotes survival during *S. aureus* bacteremia

**Figure 2:**
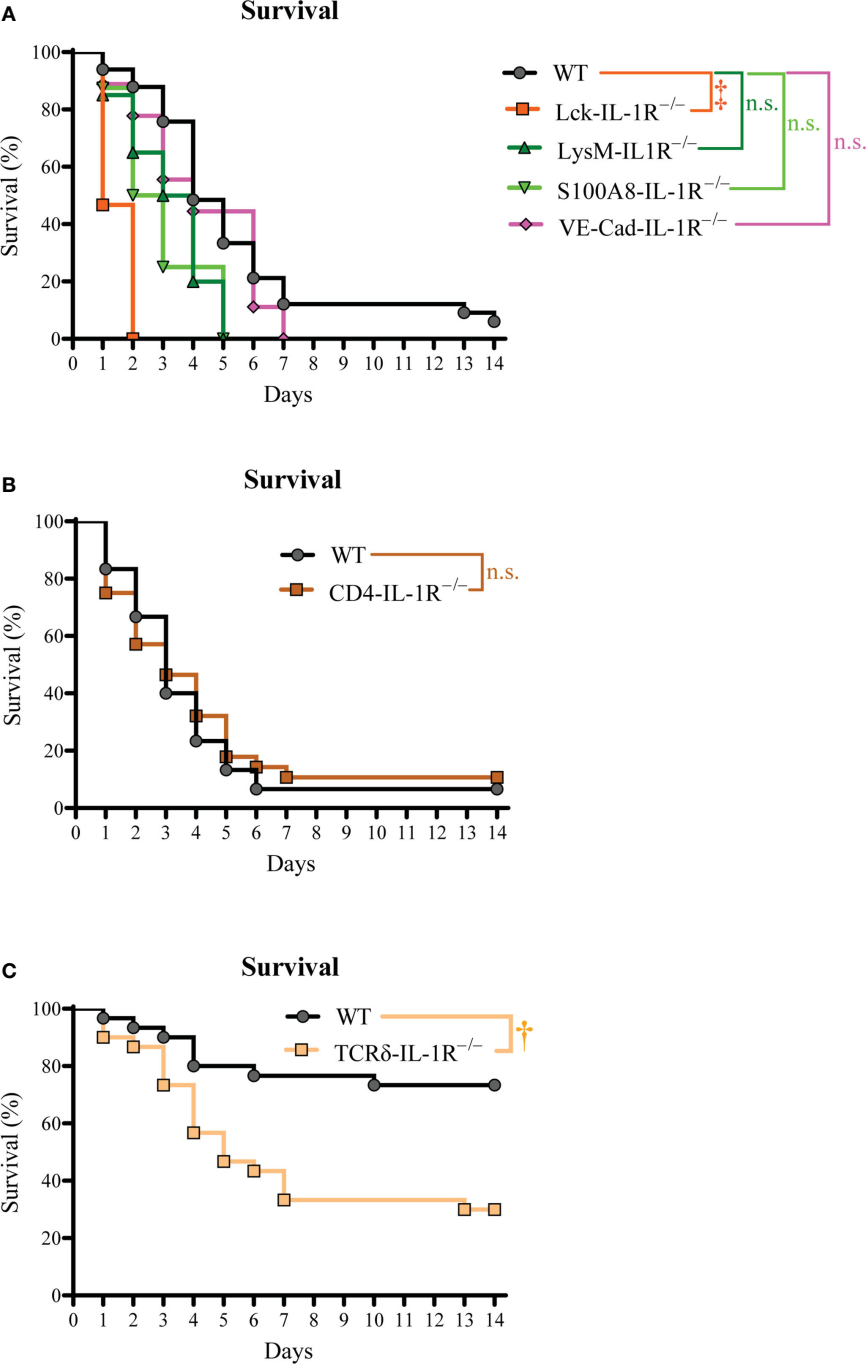
γδ T cell-intrinsic IL-1R signaling promotes survival during *S. aureus* bacteremia. The *S. aureus* bacteremia infection was performed on Lck-IL-1R^–/–^, LysM-IL-1R^–/–^, S100A8-IL-1R^–/–^, VE-Cad-IL-1R^–/–^, CD4-IL-1R^–/–^, and TCRδ-IL-1R^–/–^ mice. **(A)** Survival (%) of Lck-IL-1R^–/–^, LysM-IL-1R^–/–^, S100A8-IL-1R^–/–^, VE-Cad-IL-1R^–/–^ mice (n=8-35 per group, average inoculum = 5.4 × 10^7^ CFUs). **(B)** Survival (%) of WT and CD4-IL-1R^–/–^ mice (n=28-30 per group, average inoculum = 5.4 × 10^7^ CFUs). **(C)** Survival (%) of WT and TCRδ-IL-1R^–/–^ mice (n=29 per group, average inoculum = 5.4 × 10^7^ CFUs). †*P* < 0.01, ‡*P* < 0.001, and n.s. = not significant; WT versus Lck-IL-1R^–/–^, LysM-IL-1R^–/–^, S100A8-IL-1R^–/–^, VE-Cad-IL-1R^–/–^, CD4-IL-1R^–/–^, TCRδ-IL-1R^–/–^ as calculated by log rank (Mantel-Cox) test. Data were combined from at least 2 independent experiments.

Since IL-1R signaling was important for survival during *S. aureus* bacteremia infections, we next elucidated the specific cell types involved in the IL-1R response. Various cell types use IL-1R signaling to drive host defense and inflammation (20), including myeloid cells, T cells, and non-immune cells (21). Thus, we developed mice with specific deletion of IL-1R in T cells (Lck-IL-1R^–/–^), myeloid cells (LysM-IL-1R^–/–^), and neutrophils (S100A8-IL-1R^–/–^). We also used mice with specific deletion of IL-1R in endothelial cells (VE-Cad-IL-1R^–/–^), since *S. aureus* interacts with endothelial cells upon bacteremia infections (22). We discovered that only the Lck-IL-1R^–/–^ mice had a significant defect in survival compared to WT mice (**Figure 2A**), suggesting that IL-1R signaling on T cells, but not myeloid cells, neutrophils, or endothelial cells was important for host survival.

**Figure 3:**
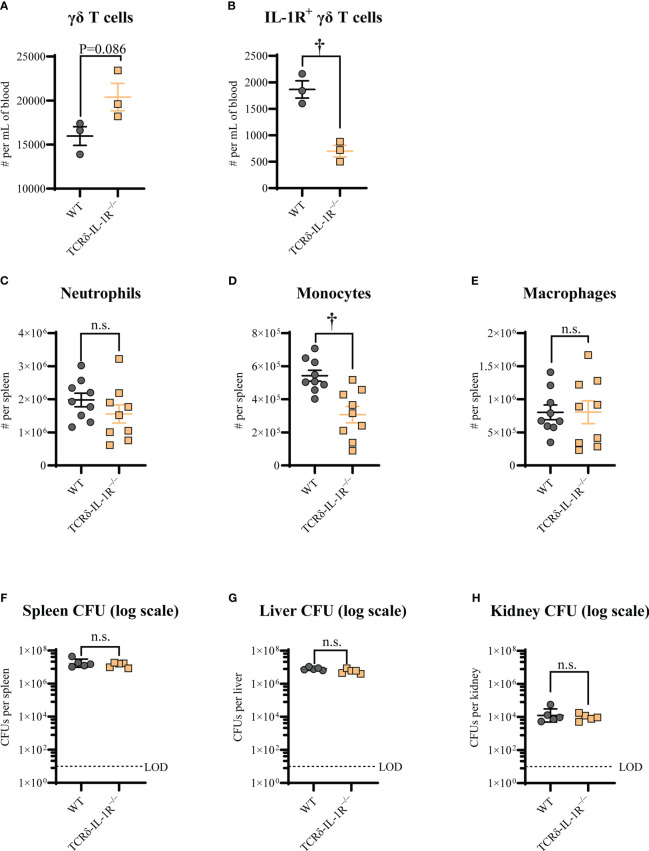
γδ T cell-intrinsic IL-1R signaling promotes monocyte recruitment during *S. aureus* bacteremia. WT and TCRδ-IL-1R–/– mice were treated daily with 100 μl of 1 mg/ml tamoxifen in sunflower oil injected intraperitoneally for 5 consecutive days. The bacteremia infections were performed 10 days after the last tamoxifen injection. After 3h post-infection, peripheral blood and spleen were collected for flow cytometry analysis, spleen, liver, and kidney were collected for ex vivo CFU enumeration. Mean cell numbers ± SEM for total γδ T cells **(A)**, IL-1R^+^ γδ T cells **(B)**, neutrophils **(C)**, monocytes **(D)**, and macrophages **(E)**. Ex vivo CFU (geometric mean ± geometric SD) for spleen **(F)**, liver **(G)**, and kidney **(H)**. n=3 per group for A and B, n=9 per group for C, D, and **(E)** n=5 per group for F, G, and **(H)** †P < 0.01 and n.s. = not significant; WT versus TCRδ-IL-1R–/– as calculated by Student’s t test. Data are combined or representative from 2 independent experiments.

We next determined the specific T cell subset required for IL-1R signaling, since CD4+ and γδ T cells are reported to be involved in host defense against *S. aureus* infections (7, 23–25). To this end, we developed and tested mice with specific deletion of IL-1R in CD4+ T cells (CD4-IL-1R^–/–^) and tamoxifen-inducible deletion of IL-1R in γδ T cells (TCRδ-IL-1R^–/–^). We discovered that CD4-IL-1R^–/–^ mice had no difference in survival compared to WT mice (**Figure 2B**). Interestingly, there was markedly decreased survival in TCRδ-IL-1R^–/–^ mice compared to WT mice (**Figure 2C**). There was a trend towards increased circulating γδ T cells counts in TCRδ-IL-1R^–/–^ mice compared to WT mice (**Figure 3A**). We confirmed tamoxifen-inducible deletion of IL-1R on γδ T cells in the TCRδ-IL-1R^–/–^ mice by flow cytometry (**Figure 3B**). Collectively, IL-1R signaling on γδ T cells was important for survival during *S. aureus* bacteremia infections.

### 3.3 γδ T cell-intrinsic IL-1R signaling increases monocyte recruitment to the spleen during *S. aureus* bacteremia

We next elucidated whether γδ T cell-intrinsic IL-1R signaling affected immune cell levels and *S. aureus* burden during the bacteremia. To this end, we first measured neutrophil, monocyte, and macrophage population levels in the spleens of TCRδ-IL-1R^–/–^ and WT mice 3 hours post-infection. We found that monocytes, but not neutrophils or macrophages, were significantly decreased in TCRδ-IL-1R^–/–^ mice compared to WT mice (**Figures 3C-E**). Next, we measured *S. aureus* CFUs in the spleen, liver, and kidney, but found no difference in bacterial burden between TCRδ-IL-1R^–/–^ and WT mice (**Figures 3F-H**). These data indicated that γδ T cell-intrinsic IL-1R signaling promoted monocyte recruitment to the spleen during *S. aureus* bacteremia.

## 4 Discussion

The IL-1, IL-17, and TNF-α cytokines contribute to host defense against *S. aureus* skin and orthopedic infections (5–8). Although IL-1, IL-17, and TNF-α cytokine levels in circulation have been associated with predictive outcomes in patients with *S. aureus* bacteremia (4, 9–12), whether these cytokines mechanistically promote host survival and the cell types involved in these responses is under-investigated. Thus, we tested mice deficient in IL-1, IL-17, and TNF-α cytokines in a preclinical mouse model of *S. aureus* bacteremia and discovered that IL-1R signaling was important for host survival. Furthermore, we identified γδ T cells as the cell type that drives IL-1R-mediated host survival against *S. aureus* bacteremia. These results provide several important insights into the protective host responses during *S. aureus* bacteremia.

First, we found that IL-1R signaling contributed to host survival during *S. aureus* bacteremia, which aligns with previously published reports (26, 27). Similarly, IL-1R signaling promotes host defense against *S. aureus* skin, orthopedic, and pneumonia infections (5, 8, 28). Interestingly, we found that both IL-1α and IL-1β were important in our model, suggesting they have non-redundant roles in host survival. This may be explained by the differences in expression profiles between the cytokines. For instance, IL-1α is constitutively expressed in non-immune cell types (29), whereas IL-1β is induced (30). Moreover, IL-1α has a nuclear localization sequence that is absent in IL-1β (31), which has important implications in inflammation (32). Understanding the differential mechanisms of protection between IL-1α and IL-1β against *S. aureus* bacteremia will be the focus of future work.

We also discovered that TNF-α and IL-17A/F did not influence host survival during *S. aureus* bacteremia at the dose tested. This was unexpected, as both TNF-α and IL-17A/F drive host defense against *S. aureus* at other infection sites (*e.g.*, skin and orthopedic implants) (5–7, 33, 34). However, in a baboon model of group A streptococcal bacteremia infection, anti-TNF-α monoclonal antibody therapy improved survival outcomes (35). Similarly, heightened TNF-α production correlated with persistent rather than resolving bacteremia in patients (12). Another possibility for the lack of phenotype in TNF-α deficient mice is that lymphotoxin-α signals through the TNF-α receptors (36, 37), which may have compensated for TNF-α deficiency in our *S. aureus* bacteremia model. Although IL-17A did not improve survival outcomes during bacteremia in our model, IL-17A limits the systemic dissemination of *S. aureus* from skin infection to kidneys (38). Thus, IL-17A may be more important in the control of *S. aureus* infections in the tissue rather than protection once bacteremia has occurred. Although not analyzed in this study, there may be a role for IL-10 in the infectious process during *S. aureus* bacteremia, as this cytokine correlates with mortality in humans (11, 12). Collectively, our findings do not support a role for TNF-α and IL-17A/F in survival outcomes during *S. aureus* bacteremia in our preclinical mouse model.

We uncovered that γδ T cell-intrinsic IL-1R signaling was crucial for host survival during *S. aureus* bacteremia. Our findings may relate to prior studies on the protective role of γδ T cells and other T cells against *S. aureus* skin infections and nasal colonization (7, 8, 39–41). In contrast, IL-1R signaling on non-hematopoietic cells was critical for protection against *S. aureus* skin infections (8). Thus, these findings indicate that the protective cell type that provides the IL-1R signal against *S. aureus* infections is context-dependent. Given that IL-1R deficient mice succumbed to *S. aureus* bacteremia within 2 days, our findings suggested that γδ T cell-mediated IL-1R signaling occurs soon after infection. In fact, γδ T cells are an innate source of pro-inflammatory responses driven by IL-1 cytokines independent of T cell receptor engagement (42, 43), perhaps explaining the importance of IL-1R signaling on this T cell subset for rapid protection against *S. aureus* bacteremia infections. However, since IL-17A/F cytokines were not important for host survival herein, and γδ T cells produce IL-17 cytokines in response to IL-1R signaling (42), it begs the question of how γδ T cell-intrinsic IL-1R signaling is mediating protection against *S. aureus* bacteremia? Our findings suggested that γδ T cell-intrinsic IL-1R signaling promotes monocyte recruitment to the spleen during *S. aureus* bacteremia as a mechanism of protection. This may relate to the known role of IL-1β to induce the monocyte-recruiting chemokine, CCL2 (44, 45). Other potential explanations include γδ T cell production of antimicrobial peptides, IL-22, and neutrophil recruiting chemokines to promote host survival (46, 47), which have been associated with protection against *S. aureus* at other infection sites (13, 48). Understanding the localization and mechanism of protection of the γδ T cell-specific IL-1R response during *S. aureus* bacteremia will be part of our future interrogations.

There were limitations. For instance, our study was conducted with a single *S. aureus* strain, limiting the broader conclusions of our findings. However, other studies have tested additional *S. aureus* strains in IL-1R deficient mice or mice treated with IL-1Ra with similar results (26, 27, 49), suggesting that IL-1R-mediated survival is not specific to a single *S. aureus* strain. Moreover, we used a high inoculum of *S. aureus* in the bacteremia model (*i.e.*, LD90), which may have missed phenotypes present in a lower inoculum (*e.g.*, LD50). Another limitation to the study is the possibility that the phenotypes in our cell-specific IL-1R deficient mice are due to changes in cytokine production in IL-1α and IL-1β rather than IL-1R-specific mechanisms. The use of tamoxifen to delete IL-1R in the TCRδ-IL-1R^–/–^ mice may have influenced the immune responses upon the *S. aureus* bacteremia infection (*e.g.*, neutrophil function) (50), which was observed in **Figure 2C**. To control for these effects, we similarly treated the control WT comparison group with tamoxifen. Importantly, deletion efficiency in γδ T cells in Lck^cre^ and TCRδ^creER^ mice is ∼20% and ∼60%, respectively (18, 51), leaving the possibility that IL-1R signaling on other T cell subsets not specifically tested in this study (*e.g.*, NK T cells and MAIT cells) contributed to host survival during *S. aureus* bacteremia infections. Addressing these limitations will be performed in our future work.

Taken together, the results of this study indicate that γδ T cell-intrinsic IL-1R signaling promotes host survival during *S. aureus* bacteremia infections. Thus, IL-1R on γδ T cells may serve as a host-directed therapeutic target for the treatment of *S. aureus* bacteremia infections and potentially other antibiotic-resistant infections. Potential therapeutic strategies could include IL-1R agonism or neutralizing the IL-1R antagonist (IL-1Ra) to promote survival during *S. aureus* bacteremia. However, further studies are warranted to understand the protective mechanisms of γδ T cell-intrinsic IL-1R signaling against *S. aureus* bacteremia.

## Data availability statement

The raw data supporting the conclusions of this article will be made available by the authors, without undue reservation.

## Ethics statement

The animal study was reviewed and approved by the Johns Hopkins Animal Care and Use Committee (Protocol #: MO21M378).

## Author contributions

YW and NA conceived and designed the study. YW conducted the experiments. YW, MA, DD, and CY collected the data. YW analyzed the data. YW and NA wrote the manuscript. All authors reviewed the final version of the manuscript.

## Supplementary material

Supplementary Figure 1 Gating strategy. **(A)** The gating strategy of myeloid cells in spleen **(B)** the gating strategy of T cells in peripheral blood.
