# Supplementary figures and images for: γδ T cell-intrinsic IL-1R promotes survival during Staphylococcus aureus bacteremia
Source: Front Immunol. 2023 Jul 7;14:1171934. doi: 10.3389/fimmu.2023.1171934 (PMC10361057; doi:10.3389/fimmu.2023.1171934)

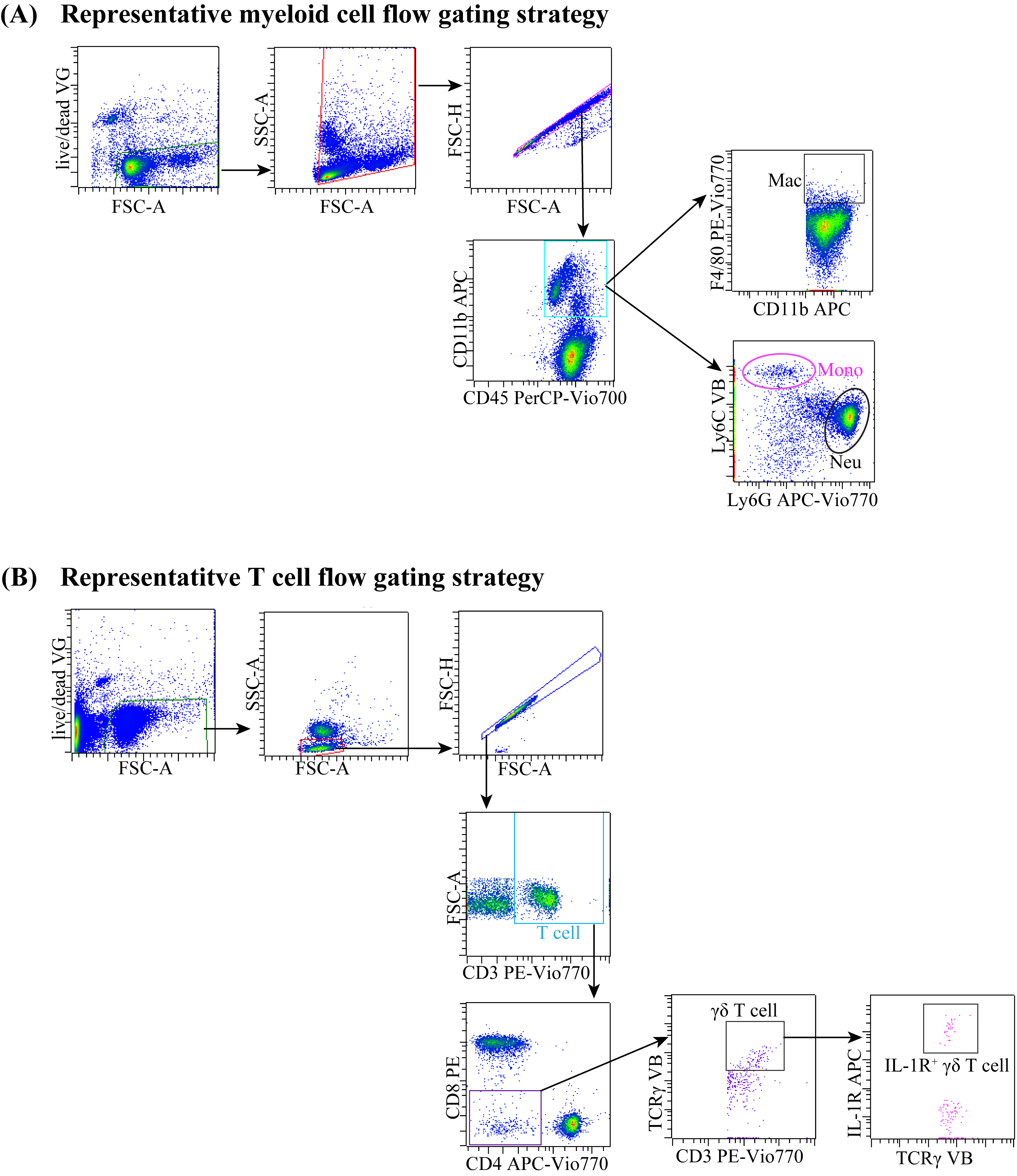

Supplement: Supplementary Figure 1 — Gating strategy. (A) The gating strategy of myeloid cells in spleen (B) the gating strategy of T cells in peripheral blood. [file Image_1.tif]
